# Maintenance of Acromegaly Control in Patients Switching From Injectable Somatostatin Receptor Ligands to Oral Octreotide

**DOI:** 10.1210/clinem/dgaa526

**Published:** 2020-08-16

**Authors:** Susan L Samson, Lisa B Nachtigall, Maria Fleseriu, Murray B Gordon, Marek Bolanowski, Artak Labadzhyan, Ehud Ur, Mark Molitch, William H Ludlam, Gary Patou, Asi Haviv, Nienke Biermasz, Andrea Giustina, Peter J Trainer, Christian J Strasburger, Laurence Kennedy, Shlomo Melmed

**Affiliations:** 1 Pituitary Center, Baylor St. Luke’s Medical Center, Baylor College of Medicine, Houston, Texas, USA; 2 Neuroendocrine Unit, Massachusetts General Hospital and Department of Medicine, Harvard Medical School, Boston, Massachusetts, USA; 3 Pituitary Center, Oregon Health & Sciences University, Portland, Oregon, USA; 4 Allegheny Neuroendocrinology Center, Allegheny General Hospital, Pittsburgh, Pennsylvania, USA; 5 Department of Endocrinology, Diabetes and Isotope Therapy, Wroclaw Medical University, Wroclaw, Poland; 6 Cedars-Sinai Medical Center, Los Angeles, California, USA; 7 University of British Columbia, Vancouver BC, Canada; 8 Northwestern University Feinberg School of Medicine, Chicago, Illinois, USA; 9 Chiasma Inc, Needham, Massachusetts, USA; 10 Leiden University Medical Center, Oegstgeest, Netherlands; 11 Institute of Endocrine and Metabolic Sciences, San Raffaele Vita-Salute University, Milan, Italy; 12 The Christie NHS Foundation Trust, Manchester, UK; 13 Clinical Endocrinology, Charite-Universitätsmedizin, Campus Mitte, Berlin, Germany; 14 Cleveland Clinic Foundation, Cleveland, Ohio, USA

**Keywords:** oral octreotide, acromegaly, IGF-1, somatostatin receptor ligands, somatostatin analogues, growth hormone

## Abstract

**Purpose:**

The phase 3 CHIASMA OPTIMAL trial (NCT03252353) evaluated efficacy and safety of oral octreotide capsules (OOCs) in patients with acromegaly who previously demonstrated biochemical control while receiving injectable somatostatin receptor ligands (SRLs).

**Methods:**

In this double-blind study, patients (N = 56) stratified by prior SRL dose were randomly assigned 1:1 to OOC or placebo for 36 weeks. The primary end point was maintenance of biochemical control at the end of treatment (mean insulin-like growth factor 1 [IGF-1] ≤ 1.0 × upper limit of normal [ULN]; weeks 34 and 36). Time to loss of IGF-1 response and proportion requiring reversion to injectable SRLs were assessed as broader control measures.

**Results:**

Mean IGF-1 measurements were 0.80 and 0.97 × ULN for OOC and 0.84 and 1.69 × ULN for placebo, at baseline and end of treatment, respectively. Mean growth hormone (GH) changed from 0.66 to 0.60 ng/mL for OOCs and 0.90 to 2.57 ng/mL for placebo. Normalization of IGF-1 levels (≤ 1.0 × ULN) was maintained in 58.2% for OOCs vs 19.4% for placebo (*P* = .008); GH levels were maintained (< 2.5 ng/mL) in 77.7% for OOC vs 30.4% for placebo (*P* = .0007). Median time to loss of response (IGF-1 > 1.0 or ≥ 1.3 × ULN definitions) for patients receiving placebo was 16 weeks; for patients receiving OOCs, it was not reached for both definitions during the 36-week trial (*P *< .0001). Of the patients in the OOC group, 75% completed the trial on oral therapy. The OOC safety profile was consistent with previous SRL experience.

**Conclusions:**

OOCs may be an effective therapy for patients with acromegaly who previously were treated with injectable SRLs.

Acromegaly is characterized by excessive circulating levels of growth hormone (GH) and insulin-like growth factor 1 (IGF-1), usually resulting from a GH-secreting pituitary adenoma (1-3). As such, GH and IGF-1 are biochemical markers of acromegaly activity (4-6). Surgical resection is the preferred primary treatment option but is not appropriate in all cases and does not result in disease control in approximately half of patients, depending on tumor size, invasiveness, and experience of the surgeon (7). For these patients, adjuvant pharmacotherapeutic treatments are indicated (8, 9).

The long-acting injectable somatostatin receptor ligands (SRLs) octreotide and lanreotide are used as first-line medical treatment for patients with acromegaly (3). However, these agents require deep tissue injection and can be associated with substantial treatment burden or deleterious long-term sequelae, including injection site pain, nodules, bruising, inflammation, and scarring (2, 10). Injectable SRLs may also negatively affect patient quality of life; patients have reported anxiety, frustration, and loss of independence caused by the injections (10, 11). Although some patients are able to safely and effectively receive injections at home, a recent study found that only 17% of patients with acromegaly are receiving injections in this manner (10, 12, 13). With the majority of SRL injections occurring in the health care delivery setting, patients report a burden to their everyday life, including impacts from travel and administration time in addition to lost work (10). A portion of patients on low doses of injectable SRLs have been found to tolerate an extended dosing interval without breakthrough symptoms (14). In a survey of real-world patient experience, approximately two-thirds of patients with acromegaly still receive the standard 28-day injectable dosing cycle (10, 14). Moreover, long-acting injectable SRLs may not provide a full month of biochemical control, and some patients report a wear-off effect with recurrence of symptoms toward the end of the dosing interval that sometimes necessitates supplemental injections (10, 15, 16).

Accordingly, oral octreotide capsules (OOCs) were developed as a potential treatment option to address challenges encountered with injectable medications (17). OOCs use a transient permeability enhancer formulation composed of an enteric-coated capsule filled with an oily octreotide suspension (18). The enteric coating facilitates the capsule to remain intact in the stomach until it traverses to the higher pH of the small intestine (18), where transient permeability enhancer excipients may enable intestinal absorption of octreotide at therapeutic levels via transitory and reversible physiologic paracellular absorption pathways (18, 19).

Registration trials evaluating currently available injectable SRLs in patients with acromegaly were primarily open-label, single-arm studies that assessed treatment efficacy based on maintenance of IGF-1 and GH levels (20-23). In a meta-analysis of clinical trials evaluating biochemical efficacy both in short- and long-acting octreotide and lanreotide formulations, overall biochemical control rates were 56% for GH and 55% for IGF-1 normalization, although there were large variations among studies (24). These results are broad assessments of acromegaly control with injectable SRLs, but direct comparisons cannot be made across studies.

Of the patients with acromegaly treated with OOC in an open-label, phase 3 study who previously responded to injectable SRLs, 65% maintained biochemical control at the end of the study and had reduced symptom severity (17). The safety profile of OOCs in that open-label trial was found to be similar to previously known safety reports for octreotide.

The CHIASMA OPTIMAL (Octreotide capsules vs Placebo Treatment In MultinationAL centers; NCT03252353) study was designed as a placebo-controlled trial to evaluate the efficacy and safety of OOCs in patients with acromegaly who previously demonstrated biochemical control on a stable long-acting injectable SRL regimen. Notably, owing to its placebo arm, the study also provides an assessment of disease activity following washout of long-term treatment, as well as distinguishing between disease-related and treatment-related adverse events (AEs) that are side effects of SRL therapy.

## Materials and Methods

CHIASMA OPTIMAL was a prospective, multicenter, randomized, double-blind, placebo-controlled study conducted under a Special Protocol Assessment–agreed protocol with the US Food and Drug Administration from August 2017 to June 2019. An institutional review board (local or central) or independent ethics committee reviewed and approved the protocol prior to study initiation, and a steering committee and an independent data monitoring committee provided study oversight. Sixty study sites were initiated, 34 of which enrolled 1 or more patients. Eligible individuals were randomly assigned 1:1 to OOCs or placebo using permuted block randomization, stratified by prior SRL dose. Written informed consent was obtained from all participants.

### Patients

The study enrolled men and women aged 18 years or older with a confirmed diagnosis of acromegaly: pituitary tumor on magnetic resonance imaging or pathology report, and documented evidence of active disease (IGF-1 ≥ 1.3 × upper limit of normal [ULN]) following 3 or more months from the most recent pituitary surgery, if performed, to ensure there was active disease prior to medical therapy. Eligible patients were required to have received long-acting injectable octreotide or lanreotide as monotherapy for 6 or more months and to be on a stable dose for 3 or more months of therapy, with biochemical control defined as IGF-1 ≤ 1.0 × ULN based on the average of 2 assessments at screening visit (SV)1 and SV2. Patients were excluded from participating if they 1) were using an off-label dose or dosing interval of a long-acting SRL injection; 2) had participated in previous OOC phase 3 clinical trials (NCT01412424 or NCT02685709); 3) had symptomatic cholelithiasis; 4) had previous conventional or stereotactic radiotherapy of the pituitary; 5) had undergone pituitary surgery within 6 months prior to screening; or 6) were treated with pegvisomant within 24 weeks, dopamine agonists within 12 weeks, or pasireotide within 24 weeks of SV1.

### Study design

**Figure 1.**
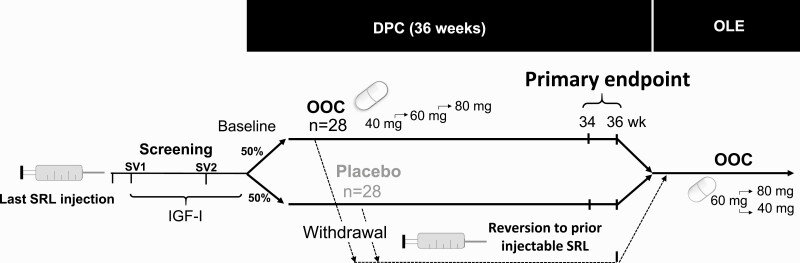
Study design for the CHIASMA OPTIMAL trial, including screening, DPC period, and OLE. DPC, double-blind placebo-controlled; IGF-1, insulin-like growth factor 1; OLE, open-label extension; OOC, oral octreotide capsules; SRL, somatostatin receptor ligand; SV, screening visit.

The study consisted of a screening period, a double-blind placebo-controlled (DPC) period and an open-label extension (OLE) (Fig. 1).

Patients were screened for study eligibility during 2 in-clinic visits (SV1 and SV2) to assess IGF-1 levels, with SV2 performed within 2 weeks of the anticipated baseline visit (4, 6, or 8 weeks from their last SRL injection). The baseline IGF-1 value, on injectable SRLs, was determined by averaging the IGF-1 values from baseline visits and SV2. Patients were started on OOCs or placebo the day they would have received their next SRL injection (± 3 days). Patients were instructed to take the capsules with water, on an empty stomach, at least 1 hour before a meal or at least 2 hours after a meal. During the 36-week DPC period, patients received 40 mg of OOC or equivalent placebo capsules—one 20-mg capsule in the morning and one in the evening. The dose was titrated at the investigator’s discretion, based on IGF-1 and/or symptoms of acromegaly, to 2 capsules in the morning and 1 capsule in the evening/night (60 mg/d) to 2 capsules twice daily (80 mg/d). Recommendations for titration included an increase of IGF-1 levels compared to baseline (defined as IGF-1 increase by ≥ 30% to > 1.0 × ULN, or IGF-1 levels > 1.0 × ULN for 2 consecutive visits), or new or worsening of acromegaly signs and symptoms. Patients entering the OLE may have received OOCs or placebo in the DPC period and were retitrated starting at 60 mg, with subsequent dose escalation or deescalation.

Patients who discontinued study treatment early for any reason reverted to their prior injectable SRL regimen and were followed until week 36. Patients were required to discontinue study drug treatment if they met predefined withdrawal criteria—defined as IGF-1 ≥ 1.3 × ULN for 2 consecutive assessments after 2 or more weeks with oral treatment on the maximum number of capsules per day (4 capsules) and exacerbation of acromegaly clinical signs/symptoms for 2 consecutive assessments. Eligible patients who completed the DPC period on OOCs or placebo or discontinued treatment after meeting the predefined withdrawal criteria were eligible to enter the OLE study and receive OOC until subsequent product approval or study termination.

### Outcomes

Efficacy outcomes for this study included the change from baseline to end of treatment in mean IGF-1 and mean GH; proportion of patients who maintained IGF-1 control (IGF-1 ≤ 1.0 × ULN) at the end of the DPC period (primary end point, determined by the average of week 34 and 36 levels for patients who completed the DPC period on study drug [discontinuation for any reason was regarded as treatment failure]); proportion of patients who maintained GH response at the end of the DPC period (secondary end point, average GH < 2.5 ng/mL, of those patients with screening GH < 2.5 ng/mL); time to loss of biochemical control in the DPC period (secondary end point, based on 2 definitions—the earliest time at which IGF-1 > 1.0 or ≥ 1.3 × ULN for 2 consecutive visits); and proportion of patients who reverted to prior injectable SRL in the DPC period (secondary end point). The main safety outcomes for this study included incidence of AEs and incidence of new or worsening AEs of special interest (AESIs) that pertained to signs or symptoms attributable to acromegaly.

### Statistics

The trial had a power of 80% or greater for various response rates assumptions on OOCs and placebo, assuming a placebo response rate of less than or equal to 10% and a difference of 40% or greater between the treatment arms. The primary efficacy analysis was based on all randomly assigned patients. The proportion of IGF-1 responders was compared between treatment groups using an exact logistic regression model with categorical covariates for treatment group, prior SRL dose, and baseline IGF-1 level. The proportion of GH responders was analyzed using the same approach, with baseline GH level instead of baseline IGF-1 level as a categorical covariate. Time to loss of response was summarized using the Kaplan-Meier method, and treatment groups were compared using a log-rank test. The proportions of patients in each treatment group who reverted to prior injectable SRL treatment prior to and including week 36 were compared using a Fisher exact test. A 2-sided 5% significance level was used for all testing, and multiplicity among the secondary end points was accounted for by testing the secondary end point in prespecified fixed testing order.

The primary end point was assessed using the nonresponse imputation (worst observation carried forward); discontinuation for any reason was regarded as treatment failure. IGF-1 and GH were summarized at each time point using descriptive statistics. Additional post hoc analyses for the primary endpoint were performed using last observation carried forward (LOCF) imputation (before initiation of injectable SRLs), as well as a completers’ analysis of response among those that completed the entire 36 weeks on the study drug.

IGF-1 and GH were measured using IDS-iSYS IGF-I (IS-3900; Immunodiagnostic Systems) (25) and IDS-iSYS hGH (IS-3700; Immunodiagnostic Systems) assays (26) at the Endocrine Laboratory, Universität München (Munich, Germany) and Solstas Lab (Greensboro, North Carolina, USA). Interassay variability was found to be 4% to 8.7% for the IGF-1 assay and 1.1% to 3.4% for GH. The assays had a sensitivity of 8.8 ng/mL for IGF-1 and 0.04 ng/mL for GH (17).

## Results

### Patient population

**Figure 2.**
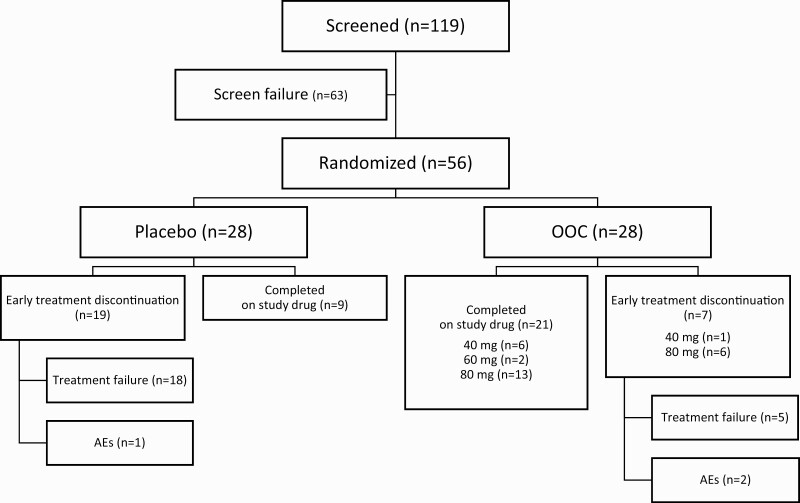
Patient disposition for all patients screened for the CHIASMA OPTIMAL trial. AE, adverse event; OOC, oral octreotide capsules.

A total of 119 patients were screened and 56 randomly assigned to receive either placebo or the study drug (Fig. 2). Sixty-three patients (52.9%) were screening failures, 73% (46/63) of whom were screening failures because of IGF-1 values greater than 1.0 × ULN while receiving injectable SRLs. All randomly assigned patients completed the DPC period per protocol. Thirty patients completed the DPC period on the study drug (OOC group, 21 patients [75.0%]; placebo group, 9 patients [32.1%]). Twenty-six patients discontinued study drug treatment during the DPC period and reverted to previous therapy (OOC group, 7 patients [25.0%]; placebo group, 19 patients [67.9%]). Discontinuation of assigned treatment was largely due to treatment failure (Fig. 2), and occurred in 18 patients in the placebo group (all of whom met the predefined withdrawal criteria) and in 5 patients in the OOC group (3 of whom met the predefined withdrawal criteria). Discontinuation due to treatment-emergent AEs (TEAEs) occurred in 2 patients in the OOC group and 1 patient in the placebo group. Treatment adherence was assessed at each study visit based on drug accountability by capsule count. Patients were compliant with the study drug dosing regimen, with a mean compliance of 98.2% in the OOC group and 96.9% in the placebo group. All patients who discontinued study drug treatment continued their participation in the study up to the end of the DPC period.

Patients were well balanced between the OOC and placebo groups (post hoc analysis showed no significant differences between groups; Table 1). All patients were receiving a long-acting parenteral SRL per label prior to inclusion in the study. Fourteen patients (50.0%) in the OOC group and 12 patients (42.9%) in the placebo group had received prior treatment with high doses of SRLs, while 8 patients (28.6%) in the OOC group and 11 patients (39.3%) in the placebo group had received prior treatment with mid doses of SRLs, and 6 patients (21.4%) in the OOC group and 5 patients (17.9%) in the placebo group had received treatment with low doses of SRLs. The symptom burden (ie, the number of acromegaly abnormalities indicated at screening) was 3 or more in 10 patients (35.7%) in the OOC group and 14 patients (50.0%) in the placebo group. The mean baseline IGF-1 was 0.80 × ULN in the OOC group and 0.84 × ULN in the placebo group.

**Table 1.** Summary of patient demographic and acromegaly baseline characteristics for the double-blind placebo-controlled period

Characteristics	OOC (n = 28)	Placebo (n = 28)	Overall (N = 56)
Sex, n (%)			
Male	12 (42.9)	14 (50.0)	26 (46.4)
Female	16 (57.1)	14 (50.0)	30 (53.6)
Race, n (%)^*a*^			
Asian	1 (3.6)	2 (7.1)	3 (5.4)
Black/African or African American	0	1 (3.6)	1 (1.8)
White	27 (96.4)	24 (85.7)	51 (91.1)
Other	0	1 (3.6)	1 (1.8)
Age at screening^*b*^, y			
Mean (SD)	55.3 (11.97)	54.2 (10.96)	54.7 (11.38)
Median	57.0	54.5	57.0
Weight at screening, kg			
Mean (SD)	83.4 (17.22)	91.6 (20.48)	87.5 (19.19)
Median	81.9	95.5	84.1
BMI at screening, kg/m^2^			
Mean (SD)	29.1 (6.26)	31.0 (5.58)	30.0 (5.96)
Median	27.8	31.2	28.8
Diabetes mellitus, n (%)	2 (7.1)	4 (14.3)	6 (10.7)
Duration of acromegaly, n (%), y			
< 10	15 (53.6)	20 (71.4)	35 (62.5)
10-< 20	8 (28.6)	5 (17.9)	13 (23.2)
≥ 20	5 (17.9)	3 (10.7)	8 (14.3)
Prior acromegaly surgery, n (%)	25 (89.3)	24 (85.7)	49 (87.5)
Symptom burden, n (%)^*c*^			
≥ 1	23 (82.1)	24 (85.7)	47 (83.9)
≥ 2	18 (64.3)	19 (67.9)	37 (66.1)
≥ 3	10 (35.7)	14 (50.0)	24 (42.9)
Screening average IGF-1, n (%)			
≤ 1.0 × ULN	28 (100)	28 (100)	56 (100)
> 1.0-< 1.3 × ULN	0	0	0
Baseline average IGF-1, n (%)^*d*^			
≤ 1.0 × ULN	27 (96.4)	23 (82.1)	50 (89.3)
> 1.0-< 1.3 × ULN	1 (3.6)	5 (17.9)	6 (10.7)
Mean baseline IGF-1 × ULN (SD)	0.8 (0.157)	0.84 (0.210)	0.82 (0.185)
Baseline GH, n (%)^*e*^			
≤ 1.0 ng/mL	23 (82.1)	21 (75.0)	44 (78.6)
> 1.0-< 2.5 ng/mL	4 (14.3)	4 (14.3)	8 (14.3)
≥ 2.5 ng/mL	1 (3.6)	3 (10.7)	4 (7.1)
Prior injectable treatment for acromegaly, n (%)			
Octreotide	19 (67.8)	17 (60.7)	36 (64.2)
Lanreotide	9 (32.1)	11 (39.3)	20 (35.7)
Prior injectable treatment overall dose, n (%)^*f*^			
Low	6 (21.4)	5 (17.9)	11 (19.6)
Middle	8 (28.6)	11 (39.3)	19 (33.9)
High	14 (50.0)	12 (42.9)	26 (46.4)

Abbreviations: BMI, body mass index; IGF-1, insulin-like growth factor 1; GH, growth hormone; OOC, oral octreotide capsules; ULN, upper limit of normal.

^*a*^Patients could have selected multiple race categories.

^*b*^Age in years = year of study day 0 – year of birth.

^*c*^Symptom burden reflects the number of ongoing acromegaly symptoms at screening on the acromegaly history form.

^*d*^Based on the average of the 2 assessments within 2 weeks prior to random assignment.

^*e*^Screening visit 1. If screening visit 1 data were missing, then baseline data were used.

^*f*^Low dose: octreotide 10 mg every 4 weeks; lanreotide 60 mg every 4 weeks or 120 mg every 8 weeks. Medium dose: octreotide 20 mg every 4 weeks; lanreotide 90 mg every 4 weeks or 120 mg every 6 weeks. High dose: octreotide 30 mg every 4 weeks; lanreotide 120 mg every 4 weeks. Patients were stratified based on the grouping of mid to high and low prior somatostatin receptor ligand doses.

### Efficacy analyses

#### Insulin-like growth factor 1 and growth hormone levels. 

**Figure 3.**
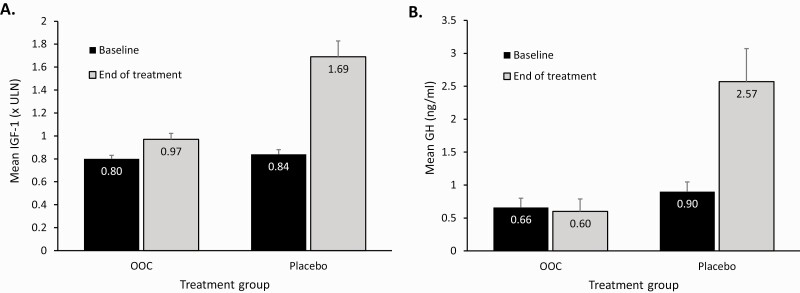
Mean levels of insulin-like growth factor 1 (IGF-1) and growth hormone (GH) at baseline and end of treatment. A, The observed mean IGF-1 change from baseline to end of DPC treatment is shown in the OOC group and the placebo group. B, The observed mean change in GH from baseline to end of DPC treatment is shown in the OOC group and the placebo group. GH levels were assessed every 30 (±5) minutes for 2 hours: at the first SV from time 0 to 2 hours (prior to SRL administration, if planned), at baseline from time 0 to 2 hours (prior to study medication), at week 36/EOT, and at all other visits in OLE, 2 to 4 hours after study medication administration. Displayed error bars show the SE. DPC, double-blind placebo-controlled; EOT, end of treatment; OOC, oral octreotide capsules; SRL, somatostatin receptor ligand; ULN, upper limit of normal.

Mean IGF-1 at baseline and at the end of study drug treatment in the OOC group was maintained within normal limits (0.80 × ULN and 0.97 × ULN, respectively). The values obtained at the end of study treatment included data in patients who discontinued treatment for any reason (including treatment failures) and excluded observations on rescue medications. In the placebo group, mean IGF-1 was within normal limits at baseline but was significantly elevated above normal limits at the end of treatment (0.84 × ULN and 1.69 × ULN, respectively) and was significantly elevated at end of treatment in comparison to the OOC group (*P* < .001; Fig. 3A). The mean GH at baseline and the end of treatment in the OOC group was 0.66 ng/mL and 0.60 ng/mL, respectively. The mean GH at baseline in the placebo group was 0.90 ng/mL but had risen to 2.57 ng/mL at the end of the treatment and was significantly elevated at the end of treatment in comparison to the OOC group (*P* = .001; Fig. 3B). At the end of the study treatment, 75% of the patients in the OOC group had IGF-1 levels of 1.1 × ULN or less.

An additional analysis of change from baseline to the end of the DPC period in IGF-1 and GH levels included data from patients who reverted to prior SRL injection. In this case, for IGF-1, the observed mean of the change from baseline to the end of the DPC period in the OOC and placebo groups was +0.13 × ULN in both cases. For GH, the observed mean of the change from baseline to the end of the DPC period in the OOC and placebo groups was –0.03 and +0.18 ng/mL, respectively. In patients reverting to the previous injectable SRL dose, the median time to return to baseline IGF-1 values following loss of response was 4.0 weeks (1 injection interval), in both the OOC (95% CI, 3.7-6.7) and placebo (95% CI, 4.0-8.3) groups.

### Study end points

**Figure 4.**
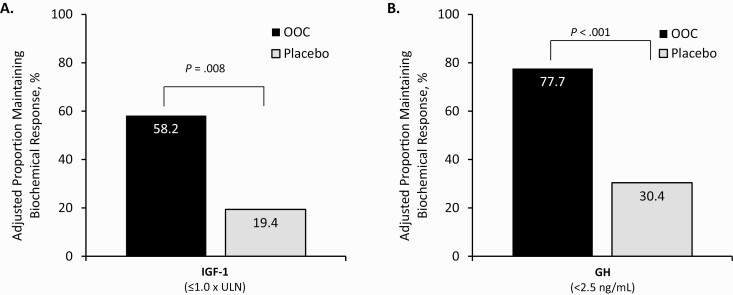
Proportion of patients who had biochemical response at the end of double-blind placebo-controlled (DPC) treatment. The proportion of patients in the OOC and the placebo groups who maintained their A, IGF-1 and B, GH response at the end of the DPC period. Adjusted proportions were obtained from an exact logistic regression model including covariates for treatment group, baseline SRL dose (low vs medium or high) and baseline IGF-I level (< median, ≥ median). GH, growth hormone; IGF-1, insulin-like growth factor 1; OOC, oral octreotide capsule; SRL, somatostatin receptor ligand; ULN, upper limit of normal.

In the OOC group, 16 of 28 participants (adjusted proportion, 58.2%; see “Materials and Methods”) met the primary end point and were biochemical responders (IGF-1 ≤ 1.0 × ULN) at the end of the DPC period compared to 5 of 28 patients in the placebo group (adjusted proportion, 19.4%) at the end of the DPC period (*P* = .008; odds ratio [OR], 5.77; 95% CI, 1.44-28.21; Fig. 4A).

Of the 12 nonresponders in the OOC group, 7 underwent early treatment discontinuation and 5 completed the DPC period on the study drug. Of these 5, 4 had IGF-1 levels of 1.1 to 1.3 × ULN, and 1 had no clinical symptoms but an IGF-1 greater than 1.3 × ULN at the end of the DPC period. Target OOC dosages in patients completing the DPC period on the study drug were 40 mg (n = 7), 60 mg (n = 2), and 80 mg (n = 19).

In the placebo group, of the 23 patients who failed to maintain biochemical response, 18 discontinued treatment because of treatment failure, 1 discontinued treatment because of a TEAE, and 4 patients completed the study on the study drug (IGF-1 level of 1.2-1.4 × ULN). Of the 5 patients in the placebo group who maintained their IGF-1 response at the week 34 and 36 visits, 3 had previously met loss of response criteria at some point during the study (2 consecutive IGF-I levels > 1.0 × ULN). All 5 patients were deemed to have active disease by their investigators (based on active symptoms and/or IGF-1 levels throughout the study) and continued on active drug in the OLE at the investigator’s discretion.

Additional post hoc analyses of the primary end point were performed for the OOC group by imputation of missing data for patients who discontinued treatment early. By imputing the last observed IGF-1 value before initiation of injectable SRLs, 18 of 28 patients (64.3%) in the OOC group were biochemical responders at the end of the study. Alternatively, analyzing proportion of responders in those completing the DPC period, 16 of 21 patients (76.2%) of patients in the OOC group were biochemical responders at the end of the study. Durability of response was analyzed by the proportion of week 24 responders (n = 12, end of dose titration) in the OOC group who maintained their response at the end of the study. Of these 12 patients, 11 (91.7%) had a sustained, durable response to end of treatment at 9 months.

In patients who previously received a low-dose of injectable SRL, 4 of 6 patients (66.7%) in the OOC group and 2 of 5 patients (40.0%) in the placebo group were responders, with an OR of 5.41 in favor of OOC. In the patients who previously received mid- to high-dose injectable SRLs, 12 of 22 patients (54.5%) in the OOC group and 3 of 23 patients (13.0%) in the placebo group were responders, with an OR of 5.86 in favor of OOC. The treatment effect was consistent in both groups (Table 2). Subgroup analysis by other characteristics showed a consistent treatment effect across age, sex, and regional location.

**Table 2.** Proportion of patients who maintained biochemical response (insulin-like growth factor 1 ≤ 1.0 × upper limit of normal) at the end of double-blind placebo-controlled treatment, by prior dose of somatostatin receptor ligand

Parameter	IGF-1 (≤ 1.0 × ULN)	
	OOC (n = 28)	Placebo (n = 28)
Overall OR (95% CI)	5.77 (1.44-28.21)	
Low prior SRL dose		
Responders, n (%)	4 (66.7)	2 (40.0)
OR (95% CI)	5.41 (0.26-165.99)	
Mid to high prior SRL dose		
Responders, n (%)	12 (54.5)	3 (13.0)
OR (95% CI)	5.86 (1.13-41.15)	

Abbreviations: IGF-1, insulin-like growth factor 1; OOC, oral octreotride capsules; OR, odds ratio; SRL, somatostatin receptor ligand; ULN, upper limit of normal.

At screening, 27 patients in the OOC group and 25 in the placebo group had mean GH values of less than 2.5 ng/mL. Of these patients, 21 of 27 patients in the OOC group (adjusted proportion, 77.7%) and 7 of 25 patients in the placebo group (adjusted proportion, 30.4%) maintained their GH response at the end of the DPC period (*P* < .001; OR 7.96; 95% CI, 2.07-36.15; Fig. 4B). An additional exploratory analysis performed in patients with GH values of less than 1.0 ng/mL at baseline showed that 65.2% of the OOC group (15/23) and 28.6% of the placebo group (6/21) maintained GH values less than 1.0 ng/mL at the end of the DPC period. Irrespective of the IGF-1 cutoff (IGF-I > 1.0 × ULN or ≥ 1.3 × ULN), the placebo group had a median time to loss of response of 16 weeks. In contrast, median time to loss of response for patients receiving OOCs was not reached during the 36-week trial (*P *< .001, both cutoffs). Seven patients (25.0%) in the OOC group vs 19 patients (67.9%) in the placebo group reverted to previous injectable SRL treatment prior to or at week 36 (*P* = .003).

### Safety analyses

Of the 56 patients in the safety population, 55 (98.2%) experienced 1 or more TEAEs during the DPC period (OOCs, 28 patients [100.0%]; placebo, 27 patients [96.4%]). Most of the TEAEs were assessed by the investigators as unrelated to the study drug. Three patients (5.4%) experienced a total of 6 TEAEs that led to study drug discontinuation (OOC, 2 patients [7.1%]; placebo, 1 patient [3.6%]) during the DPC period. TEAEs in the OOC group that led to study drug discontinuation included gastrointestinal (GI) symptoms and headache. Of patients receiving OOCs, 1 (3.6%) experienced AEs of hypoglycemia, 3 (10.7%) reported blood glucose increase, and 1 (3.6%) reported hyperglycemia.

TEAEs with an incidence of 5% or more that were more common in the OOC group than in the placebo group were diarrhea, nausea, abdominal discomfort, vomiting, dyspepsia, blood glucose increased, sinusitis, osteoarthritis, cholelithiasis, urinary tract infection, large intestine polyp, and pain (Table 3). All of the GI TEAEs reported in the OOC group were mild or moderate in intensity. The median time to onset of GI AEs was 68 days and most resolved on treatment (median duration, 8 days).

**Table 3.** Incidence of treatment-emergent adverse events occurring in 5% or more in either treatment group and more often in the oral octreotride capsules group during the double-blind placebo-controlled treatment period

System organ class preferred term	OOC (n = 28) n (%)	Placebo (n = 28) n (%)	Overall (N = 56) n (%)
Diarrhea	8 (28.6)	6 (21.4)	14 (25.0)
Nausea	6 (21.4)	3 (10.7)	9 (16.1)
Abdominal discomfort	4 (14.3)	3 (10.7)	7 (12.5)
Vomiting	4 (14.3)	0	4 (7.1)
Dyspepsia	3 (10.7)	1 (3.6)	4 (7.1)
Blood glucose increased	3 (10.7)	1 (3.6)	4 (7.1)
Sinusitis	3 (10.7)	0	3 (5.4)
Osteoarthritis	3 (10.7)	0	3 (5.4)
Cholelithiasis	2 (7.1)	1 (3.6)	3 (5.4)
Urinary tract infection	2 (7.1)	1 (3.6)	3 (5.4)
Pain	2 (7.1)	0	2 (3.6)
Large intestinal polyp	2 (7.1)	0	2 (3.6)

Treatment-emergent adverse events were defined as all adverse events (AEs) that occurred after random assignment (ie, date of onset was on or after the date of random assignment) and on or before the end of treatment (last dose of blinded study drug) in the double-blind, placebo-controlled period. A patient could be counted only once within each category. AEs were coded using *MedDRA* version 18.1.

Abbreviation: OOC, oral octreotide capsules.

AESIs that could be attributed to acromegaly were observed more frequently in patients receiving placebo than those receiving OOCs (92.9% vs 53.6%; Table 4). The most common AESIs observed were arthralgia, hyperhidrosis, headache, fatigue, carpal tunnel syndrome, and peripheral swelling.

**Table 4.** Incidence of select adverse events of special interest during the double-blind placebo-controlled period

	OOC (n = 28) n (%)	Placebo (n = 28) n (%)	Overall (N = 56) n (%)
Patients with ≥ 1 AESI	15 (53.6)	26 (92.9)	41 (73.2)
Arthralgia	7 (25.0)	15 (53.6)	22 (39.3)
Hyperhidrosis	5 (17.9)	7 (25.0)	12 (21.4)
Headache	0 (0)	9 (32.1)	9 (16.1)
Fatigue	1 (3.6)	7 (25.0)	8 (14.3)
Carpal tunnel syndrome	4 (14.3)	4 (14.3)	8 (14.3)
Peripheral swelling	3 (10.7)	4 (14.3)	7 (12.5)
Arthritis	1 (3.6)	2 (7.1)	3 (5.4)

Additional adverse events (AEs) identified by the investigator may also be included as AEs of special interest (AESIs). An individual could be counted only once within each category. AEs were coded using *MedDRA* version 18.1.

Abbreviation: OOC, oral octreotide capsules.

## Discussion

The CHIASMA OPTIMAL study met its primary and all secondary end points, demonstrating that OOCs may be an effective maintenance therapy for patients previously controlled with injectable SRLs.

In this trial, maintenance of mean IGF-1 levels for the OOC group (n = 28) from baseline to end of treatment indicates that patients who switched from injectable SRLs to OOCs retain biochemical control of acromegaly. Mean IGF-1 or GH levels at baseline and end of treatment reflects the trend in the overall treated patient population, providing clinically meaningful assessment (27, 28). Analysis of response uses stringent response cutoffs that do not take into account the fact that IGF-1 levels can be variable within a single patient (20). Transient “loss” of response does not provide a comprehensive picture of long-term and sustained disease control (5, 29).

An interesting metric of IGF-1 variability is the high screening failure rate in this study. Despite the study requirement for a “well-controlled” population (IGF-1 ≤ 1.0 × ULN), more than half of screened patients actually failed screening; the majority of these (73%) did not meet eligibility criteria, with an IGF-1 value of greater than 1.0 × ULN, confirming similar findings from a previous study (2). Normally occurring IGF-1 fluctuations in patients with acromegaly should be considered when evaluating response to therapy (30-32).

In the primary end point analysis, a significantly higher proportion of patients receiving OOCs maintained their response, as measured by IGF-1, compared to placebo (*P* = .008); this finding was also observed in the measurement of GH control. Approximately 93% of patients in the placebo group lost their response at some time during the study. Loss of response in the placebo group following withdrawal of active treatment is consistent with the literature on loss of response following withdrawal of long-acting SRLs and confirms that enrolled patients had active acromegaly (33-36). Per protocol, following the availability of study results, the pertinent study investigators were informed of the treatment allocation of the 5 placebo patients who maintained response at the end of the study. Although each of these patients met the end point of IGF-1 less than 1.0 × ULN at the end of the DPC period, 3 of these 5 patients had lost biochemical control (IGF-I > 1.0 × ULN for 2 consecutive visits) at some point during the DPC period. This finding highlights the variability of IGF-1 levels in patients with acromegaly and suggests that levels can spontaneously fluctuate and that in a few instances of untreated patients, this may result in values occasionally falling into the normal range. All 5 placebo patients continued in the OLE, providing evidence that the investigators, blinded to the randomized treatment arm, believed that these patients had active disease (clinically and/or biochemically) and would derive additional benefit from OOC treatment.

The placebo design of this study was unique for acromegaly trials, which have largely been open label. Overall, the effect between the OOC and placebo groups had an OR of 5.77 in favor of OOC. This treatment effect was not predicted by prior SRL dose (OR for patients on low-dose SRLs, 5.4; OR for patients on mid- to high-dose SRLs, 5.9), indicating that those patients with more severe disease requiring higher doses of injectable SRL also respond to OOCs.

The primary end point was assessed using the most conservative imputation of worst observation carried forward (a nonresponse imputation in which discontinuations are regarded as treatment failure) with a 58.2% adjusted response rate. Historically, imputation methods in studies of injectable SRLs have included LOCF imputation and analyses of response among completers. The former analysis allows assessment of responses in patients who discontinued study treatment early (20, 37), whereas the latter disregards these patients. Because investigation of these prior therapies used LOCF and completer analysis, results from CHIASMA OPTIMAL were also assessed post hoc, in the context of these historical imputation methods. When assessing the response rate for this population based on LOCF imputation or in study completers, the rates were 64.3% and 76.2%, respectively.

Accurate and comprehensive assessment of treatment in acromegaly should consider durability of response, as assessed by time to loss of response and sustainability of response. While the placebo group had a median time to loss of response of 16.0 or 16.2 weeks (IGF-1 > 1.0 × ULN and ≥ 1.3 × ULN, respectively), the OOC group did not reach the median time to loss of response within the 36-week DPC period for either cutoff. Ninety-two percent of patients responding to OOC at the end of the 24 weeks’ titration maintained responses at 9 months. These results provide evidence for the durability of response with OOCs, even on the stringent response cutoffs. The small proportion of patients who did not sustain a durable response was similar to that reported for studies of patients in which injectable SRL responders at baseline were followed over time (38-41).

The need of SRL uptitration to improve biochemical control is well known (42), and OOCs may have a shorter and less burdensome titration algorithm, compared to injectable SRLs, at the end of which responders can be identified; nonresponders should revert to prior treatment. In CHIASMA OPTIMAL, patients requiring reversion to prior injectable SRL treatment reestablished their baseline response level in a median of 4.0 weeks, the equivalent of a single injectable SRL administration at prestudy doses. This is also supported by IGF-1 change from baseline in the OOC group, including data in patients who reverted to injectable SRLs who showed no change and therefore no clinically relevant deterioration in IGF-1 control. This observation suggests that patients can safely be offered a trial therapy with OOCs, and those not responding can return to prior treatment without deterioration in IGF-1 control.

Reflecting the positive experience of patients and their clinicians with OOC treatments in this trial, 90% of patients receiving OOCs at the end of the DPC period chose to remain on active treatment in an OLE phase.

The observed safety profile of OOCs was consistent with the known safety of injectable octreotide (except for lack of injection site reactions) as well as the disease burden of acromegaly. No new or unexpected safety signals were detected. The most commonly reported TEAEs were similar to those reported with long-acting injectable SRLs but with no injection site reactions. Most TEAEs were mild or moderate in intensity, and the highest incidences of TEAEs were GI related. These were mild or moderate in intensity and most resolved within a median of 8 days with continued dosing of OOCs. OOCs were well tolerated, and only 2 patients (7.1%) in the OOC group discontinued because of an AE.

The placebo-controlled design of this study also allowed for assessment of AEs that may occur owing to active symptoms of acromegaly. Consistent with loss of biochemical control, approximately double the AESIs were observed in the placebo group, including higher rates of arthralgia, hyperhidrosis, fatigue, carpal tunnel syndrome, and peripheral swelling.

Surgical resection of pituitary adenomas is the preferred treatment in most patients with acromegaly, but may not be curative, and many patients require medical adjuvant therapy (43), including SRLs, GH receptor blockers, and dopamine agonists as single or combination therapy (6). Although long-acting injectable SRLs are usually used as first-line medical treatment, they require deep tissue injections and can be associated with numerous side effects and lifestyle burdens (6, 10, 44). Most patients report persistent or recurrent injection site pain, local nodules, and swelling as well as bothersome breakthrough acromegaly symptoms occurring toward the end of the injection cycle (45). These untoward patient complaints highlight the need for an alternative treatment (10). Oral octreotide was developed to address these unmet needs. This pivotal placebo-controlled study indicates that OOCs may be an effective option in patients with acromegaly currently responding to injectable SRLs, while potentially avoiding side effects and compliance issues related to injectable regimens.

## Data Availability

The data sets generated during and/or analyzed during the present study are not publicly available but are available from the corresponding author on reasonable request.

## Abbreviations:

AE: adverse event
AESI: adverse event of special interest
DPC: double-blind placebo-controlled
GH: growth hormone
GI: gastrointestinal
IGF-1: insulin-like growth factor 1
LOCF: last observation carried forward
OLE: open-label extension
OOC: oral octreotide capsule
SRL: somatostatin receptor ligand
SV: screening visit
TEAE: treatment-emergent adverse event
ULN: upper limit of normal

## Additional Information

***Disclosure Summary:*** S.S., A.G., and S.M. are Chiasma advisory board members. S.S. and M.B. are Novartis advisory board members. L.N. and M.B. are Pfizer advisory board members. M.B. is a Merck advisory board member. P.T. is a MidaTech (previously Ono) advisory board member. L.K. is a Corcept and Novo Nordisk advisory board member. L.N., A.G., M.F., and C.S. are consultants for Pfizer. M.F., C.S., and S.M. are consultants for Chiasma. M.F., C.S., and S.M. are consultants for Crinetics. M.F., A.G., and C.S. are consultants for Ipsen. M.F. and S.M. are consultants for Ionis. M.F and M.M. are consultants for Novartis. M.G. is a consultant for Corcept. M.G., M.M., and C.S. are consultants for Novo Nordisk. M.B. is a consultant for Pfizer Poland, Novartis Poland, Ipsen Poland, and Merck Poland. M.M. is a consultant for Abbott, AstraZeneca, Bristol-Myers Squibb, Eli Lilly, Janssen, and Merck. A.G. is a consultant for Genevant. C.S. is a consultant for Ascendis, Strongbridge, Merck Serono, Serono, and Tiburio. S.M. is a consultant for Rami. S.S., M.G., and A.G. have received grants from Novartis. L.N. and M.G. have received grants from Chiasma. L.N., A.G., and M.G. have received grants from Ipsen. M.G. and L.K. have received grants from Novo Nordisk. M.G. and C.S. have received grants from Crinetics. M.G. has received grants from Camurus, Corcept, OPKO, and Teva. M.M. has received grants from Amgen and Sanofi. A.G., C.S., and S.M. have received grants from Pfizer. C.S. has received grants from Aeterna Centaris, Centaris, and Tuburio. S.S., A.L., L.K., L.N., M.B., M.F., M.G., and P.T. are research investigators for Chiasma. S.S. is a research investigator for Corcept and OPKO. S.S., P.T., M.M., and M.F. are research investigators for Novartis. M.F., L.K., and P.T. are research investigators for Crinetics. M.F., L.K., and A.L. are research investigators for Ionis. M.M. is a research investigator for Bayer, Reata, and Tercica. M.M. and L.K. are research investigators for Novo Nordisk. L.K. is a research investigator for Strongbridge. P.T. is a research investigator for Camarus and Tiburio. P.T. and S.M. are research investigators for Ipsen. W.L., G.P., and A.H. are employees of Chiasma. L.N. and C.S. are speakers for Ipsen. M.M. is a speaker for Abbott, Sanofi-Aventis, and Merck. C.S. is a speaker for Novo Nordisk, Pfizer, and Sandoz. L.K. is a speaker for Corcept. The spouse of M.M. has ownership interest in Amgen. E.U. and N.B. have nothing to disclose.
